# A novel homozygous frameshift mutation in *CFAP65* is associated with multiple morphological abnormalities of sperm flagella in a consanguineous Pakistani family

**DOI:** 10.1186/s12610-026-00319-z

**Published:** 2026-07-20

**Authors:** Musavir Abbas, Ansar Hussain, Ma Hui, Haider Ali, Muhammad Lateef, Mujahid Hussain, Ghulam Mustafa, Baolu Shi, Huan Zhang, Tingting Lin, Hao Yin, Qinghua Shi

**Affiliations:** 1 Centre for Reproduction and Genetics, Department of Obstetrics and Gynecology, First Affiliated Hospital of USTC, Hefei National Laboratory for Physical Sciences at Microscale, School of Basic Medical Sciences, Biomedical Sciences and Health Laboratory of Anhui Province, Institute of Health and Medicine, Hefei Comprehensive National Science Centre, Division of Life Sciences and Medicine, University of Science and Technology of China, Hefei, China; 2 Chongqing Key Laboratory of Human Embryo Engineering, Center for Reproductive Medicine, Women and Children’s Hospital of Chongqing Medical University, Chongqing, China

**Keywords:** Male infertility, *CFAP65*, MMAF, Flagellar defects, Axonemal disorganization, Infertilité masculine, *CFAP65*, MMAF, Anomalies flagellaires, Désorganisation axonémale

## Abstract

Multiple morphological abnormalities of the sperm flagella (MMAF) represent a severe form of asthenozoospermia that leads to male infertility, and it is commonly associated with genetic defects affecting flagellar components. Although the gene *CFAP65* has been implicated in MMAF, its full mutational spectrum and clinical relevance within highly consanguineous populations remain poorly characterized. To elucidate the genetic basis of this condition, whole-exome sequencing followed by Sanger sequencing was performed in two infertile individuals from a consanguineous Pakistani family. Sperm morphology was assessed using hematoxylin-eosin (H&E) staining; while scanning and transmission electron microscopy (SEM and TEM) were utilized to evaluate ultrastructural defects. A novel homozygous frameshift mutation in *CFAP65* (c.582_587delinsCG; p.Q194Hfs*4) was identified. This mutation introduces a premature stop codon within the transmembrane helix domain. Morphological analysis revealed classic MMAF features, including absent, short, bent, and coiled flagella in over 97% of sperm. TEM further demonstrated severe axonemal disorganization, with absent or disrupted microtubule doublets observed in 85% of the cross-sections. Based on clinical history and chest X-ray, the affected individuals reported no chronic respiratory symptoms and had no situs inversus, suggesting an isolated sperm-specific phenotype. In conclusion, this study identifies a novel domain-specific *CFAP65* mutation associated with MMAF and male infertility in a consanguineous Pakistani family, thereby expanding the known genetic mutational landscape of MMAF. These findings reinforce the crucial role of *CFAP65* in sperm flagellar morphogenesis and underscore its significance in the genetic diagnosis of male infertility.

**Supplementary Information:**

The online version contains supplementary material available at 10.1186/s12610-026-00319-z.

## Résumé

Les anomalies morphologiques multiples du flagelle spermatique (MMAF) représentent une forme sévère d’asthénozoospermie conduisant à l’infertilité masculine, fréquemment associée à des anomalies génétiques affectant les composants flagellaires. Bien que le gène *CFAP65* ait été impliqué dans la MMAF, son spectre mutationnel complet et sa pertinence clinique au sein de populations hautement consanguines demeurent mal caractérisés. Afin d’élucider la base génétique de cette affection, un séquençage de l’exome entier suivi d’un séquençage Sanger a été réalisé chez deux individus infertiles issus d’une famille pakistanaise consanguine. La morphologie des spermatozoïdes a été évaluée par coloration à l’hématoxyline-éosine (H&E), tandis que la microscopie électronique à balayage et à transmission (MEB et MET) a permis d’analyser les défauts ultrastructuraux. Une nouvelle mutation homozygote entraînant un décalage du cadre de lecture dans *CFAP65*(c.582_587delinsCG ; p.Q194Hfs*4) a été identifiée. Cette mutation introduit un codon stop prématuré au sein du domaine de l’hélice transmembranaire. L’analyse morphologique a révélé les caractéristiques classiques de la MMAF, incluant des flagelles absents, courts, angulés et enroulés dans plus de 97% des spermatozoïdes. La MET a en outre mis en évidence une désorganisation axonémale sévère, avec absence ou altération des doublets de microtubules observée dans 85% des coupes transversales. Sur la base de l’histoire clinique et de la radiographie thoracique, les individus affectés ne présentaient aucun symptôme respiratoire chronique ni situs inversus, suggérant un phénotype isolé spécifique aux spermatozoïdes. En conclusion, cette étude identifie une nouvelle mutation de *CFAP65* spécifique d’un domaine, associée à la MMAF et à l’infertilité masculine dans une famille pakistanaise consanguine, élargissant ainsi le paysage mutationnel connu de la MMAF. Ces résultats renforcent le rôle crucial de *CFAP65*dans la morphogenèse du flagelle spermatique et soulignent son importance dans le diagnostic génétique de l’infertilité masculine.

## Background

Multiple morphological abnormalities of the sperm flagella (MMAF) represent a severe form of asthenozoospermia, characterized by aberrant sperm flagellar structures-including bent, coiled, irregular, short, or absent flagella-and resulting in compromised motility and male infertility [1–5]. Ultrastructural analyses commonly reveal axonemal disorganization, including absence of the central pair complex and disrupted mitochondrial sheath [3, 6, 7].

Recent advances in next-generation sequencing have enabled the identification of numerous genetic causes underlying MMAF. To date, pathogenic variants have been reported in several cilia-and flagella-associated proteins (*CFAPs*), including *CFAP43*,* CFAP44*,* CFAP65*,* CFAP69*,* CFAP70*, and *CFAP251* [1, 4, 8–13]. These genes function in distinct flagellar compartments: *CFAP43*, *CFAP44*, and *CFAP65* are associated with the inner dynein arm tether/tether head (IDA-T/TH) complex; *CFAP69* with intraflagellar transport [14]; *CFAP70* with the outer dynein arm (ODA)-associated complex; and *CFAP251* with the calmodulin and spoke-associated complex (CSC) [15]. Among them, *CFAP43* and *CFAP44* account for approximately 8-31% of MMAF cases across various populations, with causality confirmed in corresponding mouse models [1, 9, 10].

Within Pakistani populations, several MMAF-associated mutations have been identified. Biallelic loss-of-function mutations in *CFAP43*, *CFAP61*, *CFAP58*, and *DNHD1* have been reported in consanguineous Pakistani families, with transmission electron microscopy and mouse models confirming pathogenicity [16–19]. The Pakistani population, characterized by a high prevalence of consanguineous marriages (approximately 65%), represents a genetic hotspot for the discovery of rare autosomal recessive mutations [20].

*CFAP65* (also known as *CCDC108* and *AKAP240*) spans 35 exons on chromosome 2q35 and encodes a 1925-amino acid transmembrane protein with two predicted functional domains, ASH (ASPM-SPD2-Hydin) and MSP (Major Sperm Protein). The ASH domain is commonly found in proteins localized to cilia, flagella, centrosomes, and the Golgi apparatus, implicating its role in microtubule-based organelle organization primarily functioning as a protein-protein interaction module, contributes to the assembly of dynamic cytoskeletal structures and mediates intracellular signaling [21]. *CFAP65* is predominantly expressed in the testis and has been shown to be essential for the biogenesis and motility of sperm flagella. Previous studies have linked *CFAP65* mutations to asthenozoospermia and MMAF across species, including chickens, humans, and mice [22, 23].

In this study, we report a consanguineous Pakistani family with two infertile males exhibiting the MMAF phenotype. Whole-exome sequencing followed by Sanger sequencing identified a novel homozygous frameshift mutation in *CFAP65* (c.582_587delinsCG) located in exon 4. The variant substitutes the glutamine (Q) residue at position 194 with a histidine (H) residue within the predicted transmembrane helix domain (187-209 aa), and leads to severe axonemal disorganization characterized primarily by disruption of the microtubule doublets while preserving acrosome. To our knowledge, this is the first report linking a *CFAP65* frameshift mutation to MMAF in a Pakistani cohort, with relatively isolated axonemal defects. These findings expand the mutational and phenotypic spectrum of *CFAP65*-related male infertility and provide important insights for genetic diagnosis and counseling in affected populations.

## Materials and methods

### Study subjects and clinical investigation

A consanguineous Pakistani family with two infertile male members (II:3 and II:5) diagnosed with asthenozoospermia was recruited for this study. Family pedigree has been presented. Routine semen analyses were performed on both patients P1 (II:3) and P2 (II:5) in accordance with the World Health Organization standard guidelines [24].

This study was approved by the Ethics Committee of the First Affiliated Hospital of the University of Science and Technology of China (USTC) (Approval ID: 2019-KY-168). Written informed consent was obtained from all participants before the study commenced.

### Hematoxylin and Eosin (H&E) staining

Sperm morphology in both patients, P1 (II:3) and P2 (II:5), was evaluated using H&E staining according to the WHO standard guidelines [24]. Briefly, patient smear slides were stained with hematoxylin for 25 min, then rinsed with double-distilled water (ddH₂O) for 1 min. The slides were immersed in 1% HCl and washed with tap water to remove excess hematoxylin. Next, the slides were sequentially dehydrated in 50%, 70%, and 80% ethanol for 1 min each, stained with eosin for 5 min, and then further dehydrated in 100% ethanol for 2 min. After staining, the slides were immersed in xylene for 5 min and mounted with coverslips using natural balsam. Morphological analysis was conducted using an optical microscope (Nikon, Tokyo, Japan) to examine at least 200 spermatozoa on each smear slide from the patients quantified and presented.

### Whole-exome sequencing and variant screening

Genomic DNA was extracted from the peripheral blood cells of the family members (II:1, II:3, and II:5) using the Flexi Gene DNA Kit, following the manufacturer’s instructions. DNA samples from patients II:3 and II:5, along with their control brother (II:1), were subjected to WES using the AIExome Enrichment Kit V1 and HiSeq2000 platforms, as per standard protocols. The filtered reads were mapped to the human reference genome (hg19) using Burrows-Wheeler Alignment software [25]. SAM files generated from the mapping were converted into BAM files using SAMtools (http://samtools.sourceforge.net/). PCR duplicates were removed, and properly paired reads were retained using Picard software (http://picard.sourceforge.net/). The processed BAM files were analyzed with ANNOVAR [26] and the Genome Analysis Toolkit (GATK) [27] (http://www.broadinstitute.org/gatk/). Indel realignment was performed using GATK’s indel realigner, and single-nucleotide variants (SNVs), small insertions, and deletions within coding sequence intervals were identified using the GATK Unified Genotyper.

Variants following a recessive inheritance pattern were filtered based on previously established methods. The bioinformatics pipeline used in this filtering process is detailed in the Supporting Information. BCF tool was employed to identify runs of homozygosity [28]. Runs of homozygosity longer than 1.5 Mb were used to calculate inbreeding coefficients, utilizing an in-house script. The relatedness of family members was validated using the Peddy tool [29]. The *CFAP65* genomic sequence was retrieved from Ensembl (https://grch37.ensembl.org/index.html) and analyzed through multiple sequence alignment with Clustal Omega. The functional impact of SNVs was assessed using PolyPhen-2, CADD, SIFT, and MutationTaster software.

Finally, Sanger sequencing was performed using the following primers for *CFAP65*: forward primer 5′-CATCTCCTGATCGCTTTCTC-3′ and reverse primer 5′-TAATATCGCAGCCTTTACCC-3’ were used.

### Scanning electron microscopy (SEM) analysis

Due to the limited number of samples, only patient P2 (II:5) semen samples were prepared for SEM analysis based on the previous protocol [30]. Spermatozoa were fixed in 2.5% phosphate-buffered glutaraldehyde at 4 °C for 2 h. The immobilized spermatozoa were deposited on poly-L-lysine-coated coverslips, washed in distilled water or PBS, and dehydrated through an ascending gradient of cold ethanol (30%, 50%, 70%, 80%, 90%, and 100%). The samples were then dried at the critical point using a Leica EM CPD300 Critical Point Dryer (Leica). Subsequently, the specimens were mounted on specimen holders and coated with gold particles using an ion sputter coater (EM ACE200, Leica). Imaging was performed using an S-3400 N scanning electron microscope (Hitachi, Tokyo, Japan).

### Transmission electron microscopy analysis

For ultrastructure analysis, spermatozoa from patient P2 (II:5) were examined using TEM following a standard methodology [31, 32]. Spermatozoa were first washed in PBS and then fixed overnight in 0.1 mol L-1 cacodylate buffer (with a pH = 7.4) containing 8% glutaraldehyde, 4% paraformaldehyde, and 0.2% picric acid. A Hitachi S-4800 Field Emission Scanning Electron Microscope (Hitachi, Tokyo, Japan) was used for the analysis, and it was accelerated to 15 kV. Selected spermatozoa were preserved in 1% OsO4 after four washes in 0.1 mol L-1 cacodylate solution in preparation for transmission electron microscopy [33]. The samples were then permeated with a combination of acetone and resins after being dehydrated in a graded sequence of ethanol concentrations (30%, 50%, 75%, 95%, and 100%). Following their embedding in paraffin, the specimens were sectioned into incredibly thin slices (70 nm) and stained with uranyl acetate and lead citrate. The flagellar ultrastructure was viewed and examined using a Hitachi transmission electron microscope operating at 100 kV or a Philips CM10 microscope (Philips Electronics, Eindhoven, The Netherlands) operating at 100 kV.

### In silico analysis

To investigate the functional predictions of the identified frameshift mutation (c.582_587delinsCG, p.Q194Hfs*4), the genomic sequence of *CFAP65* (ENST00000341552) was retrieved from the Ensembl Genome Browser 37 (http://grch37.ensembl.org/). Bioinformatics tools were used to assess the potential effect of the *CFAP65* variant, such as MutationTaster2, and Franklins ACMG (https://www.genecascade.org/MutationTaster2025/), (https://franklin.genoox.com/).

### Statistical analysis

Statistical analysis was performed using a student’s t-test. Results are expressed as the mean ± SEM. A significance level of *P* < 0.05 was considered statistically significant, with * denoting *P* < 0.05.

## Results

### Identification of a novel homozygous *CFAP65* frameshift mutation in a consanguineous Pakistani family

**Fig. 1:**
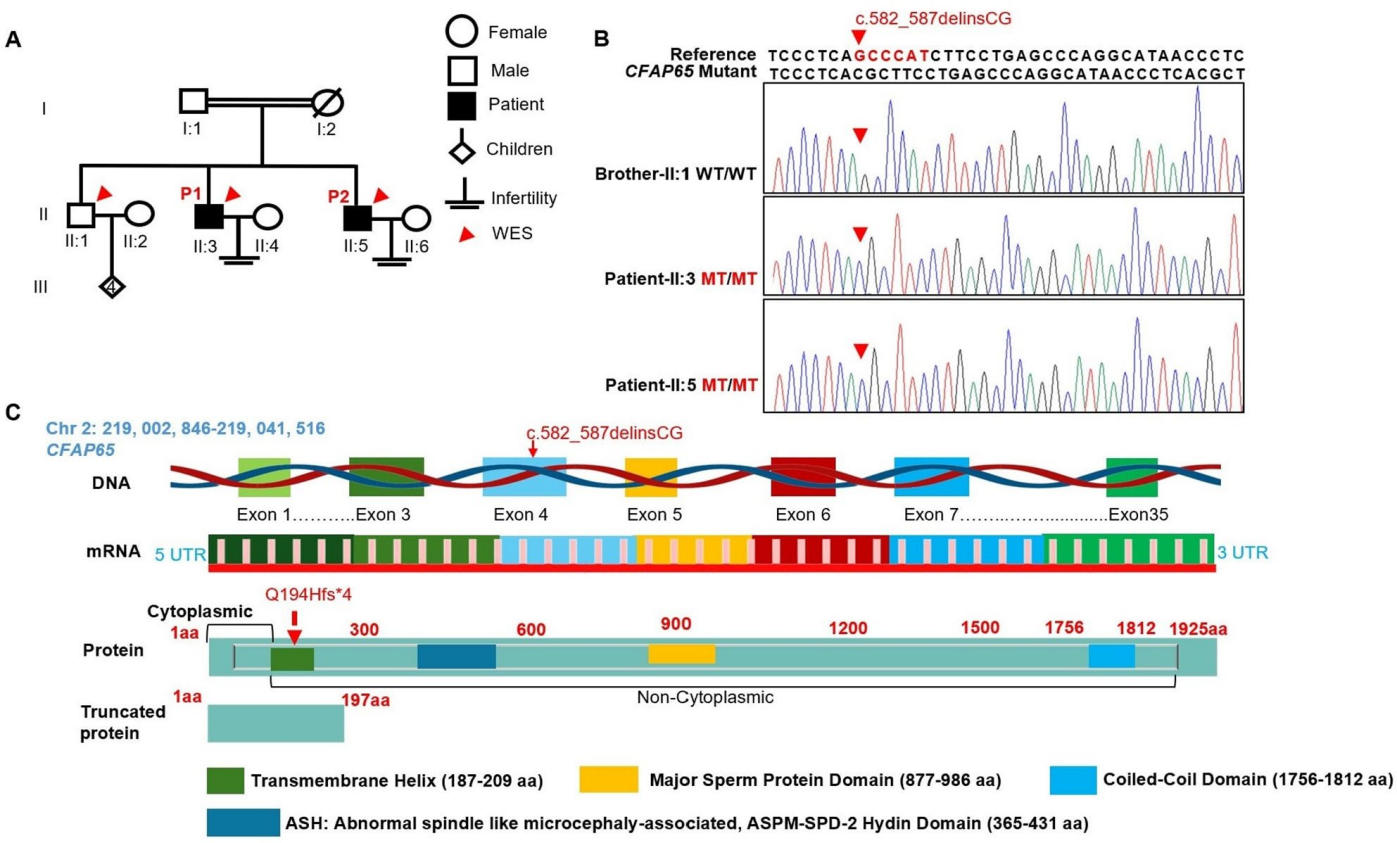
Identification of a homozygous frameshift variation in *CFAP65* from a Pakistani consanguineous family. **A** Pedigree of the studied family. Whole-exome sequencing was performed on the individuals indicated by red arrowheads. Double horizontal lines indicate consanguineous marriages. Squares and circles denote male and female, respectively. Solid symbols denote infertile males, open symbols represent unaffected individuals and slashes indicate deceased members. **B** Sanger sequencing validation of the *CFAP65* mutation in available family members. The red arrowhead indicates the mutation site. **C** Schematic representing of the *CFAP65* frameshift mutation at genomic and protein levels. The identified variant leads to a frameshift within the transmembrane helix domain (187–209 aa), resulting in the substitution of glutamine “Q” by histidine “H” at position 194. Location of *CFAP65* mutation in CFAP65 protein indicated with red arrow. Dark green square represents the transmembrane helix region; turquoise square represents the ASPM-SPD-2-Hydin (ASH) domain; gold square represents the major sperm protein (MSP) domain and blue square represents the coiled-coil region

This study investigated a consanguineous Pakistani family with idiopathic male infertility (Fig. 1A). The two affected brothers (P1: II:3 and P2: II:5), born to first-cousin parents, married at ages 25 and 29, respectively, corresponding to marriage durations of 12 and 10 years (Table 1). Both patients exhibited normal pubertal development, secondary sexual characteristics, and maintained physiological levels of reproductive hormones (follicle-stimulating hormone and testosterone). Chromosomal analysis revealed normal karyotypes (46, XY) with no evidence of Y chromosome microdeletions. Semen analyses revealed normal ejaculate volume and sperm concentration, but markedly reduced sperm motility (10 ± 1.16 and 18.67 ± 2.6, respectively; Table 1), consistent with asthenozoospermia. Neither brother reported clinical symptoms of primary ciliary dyskinesia (PCD). Chest radiographs showed no abnormalities suggestive of PCD, such as bronchiectasis, atelectasis, or chronic infiltrates (Supplementary Figure S2). Their spouses had unremarkable gynecological histories, including regular menstrual cycles and no evidence of miscarriages or ovarian dysfunction.

**Table 1:** Semen parameters of the patients carrying the homozygous *CFAP65* frameshift mutation

	Reference values	P1 (II:3)	P2 (II:5)
Physical examination^a^		-	-
Fertility state	-	Infertile	Infertile
Age (years old) ^b^	-	37	39
Years of marriage	-	12	10
Height/weight (cm/kg)	-	165/64	170/72
Genetic testing			
Karyotype	-	46, XY	46, XY
Y-chromosome microdeletion	-	Negative	Negative
Semen parameters ^c^			
Semen volume (ml)	>1.4	2.5±0.29	3±0.29
Semen pH	Alkaline	Alkaline	Alkaline
Sperm concentration (10^6^/ml)	>16.0	18.0±1.16	22.67±2.6
Motile sperm (%)	>42.0	10±1.16	18.67±2.4
Immotile sperm (%)		90±1.16	81.33±2.5
Flagella ^d^			
Normal (%)	>23.0	3.067±0.067	2.67±0.33
Coiled (%)	<17.0	11.67±0.89	12.83±0.6
Short (%)	<1.0	29.73±0.27	24.83±0.44
Bent (%)	<17.0	3.45±0.28	14.43±1.26
Absent (%)	<5.0	42.03±0.27	36.67±0.89
Irregular (%)	<2.0	11.05±0.05	8.57±0.8
Hormone concentrations^e^			
FSH (mIU/mL)	1.3-13.5	9.4	5.83
Testosterone (nmol/L)	6.4-31.8	13.75	9.48

^a^Physical examination was performed by the local andrologist

^b^ Ages at the manuscript submission

^c^ Semen analysis was performed for each infertile individual following the WHO guidelines (World Health Organization, 2021)

^d^ Three independent experiments were performed. Data are presented as mean ± SEM

^e^ Reference values were suggested by the local clinical laboratory. FSH, follicle stimulating hormone

To uncover the genetic basis of the male infertile patients from the consanguineous Pakistani family, we performed whole-exome sequencing (WES) on the two affected individuals (II:3 and II:5) and an unaffected fertile brother (II:1) (Fig. 1A). After variant filtering and Sanger sequencing of available family members, we identified a homozygous frameshift variant in *CFAP65* (c.582_587delinsCG) that co-segregated with asthenozoospermia (Fig. 1B, Supplementary Figure S1). The mutation located in exon 4 results in a frameshift beginning at codon 194, replacing glutamine with histidine and introducing a premature stop codon at position 197 (p.Q194Hfs*4), leading to a predicted truncated protein (Fig. 1C).

Autozygosity mapping confirmed that the *CFAP65* variant resides within a shared homozygous region in both affected individuals, consistent with autosomal recessive inheritance in this consanguineous pedigree (Supplementary Figure S1B). Sanger sequencing validated the mutation and demonstrated complete segregation with the disease phenotype (Fig. 1B). The variant was absent in unaffected family members and not reported in population databases (gnomAd and 1000genome), further bioinformatic tools such as MutationTaster2, and Franklins classified it to be deleterious and likely pathogenic accordingly. These findings indicate that the homozygous frameshift variant of *CFAP65* (c.582_587delinsCG: p.Q194Hfs*4: NM_194302.3) is likely pathogenic according to The American College of Medical Genetics and Genomics (ACMG) guidelines and is the likely genetic cause of asthenozoospermia and male infertility in this family.

### *CFAP65* mutation underlies MMAF-associated asthenozoospermia

**Fig. 2:**
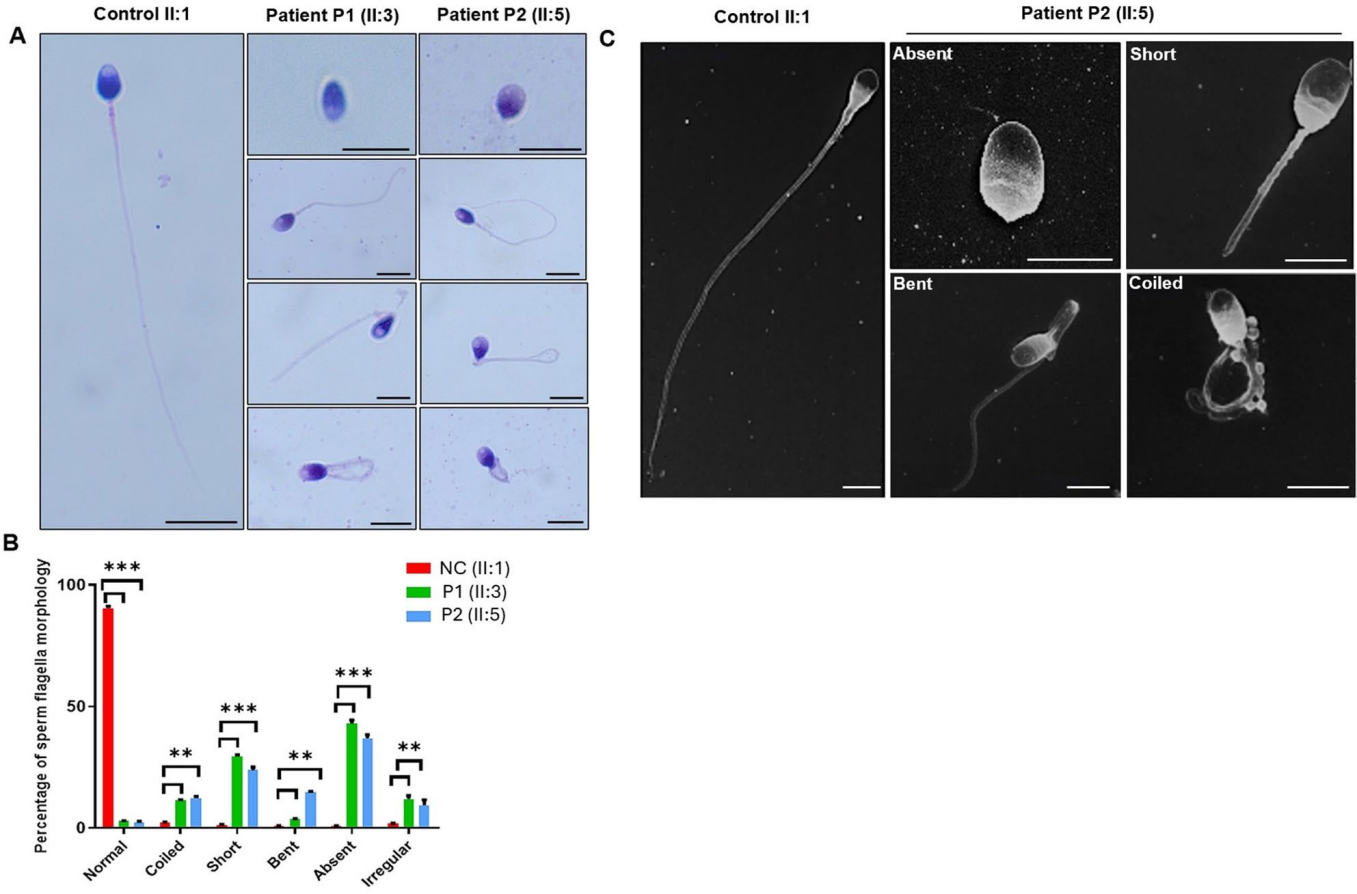
The multiple morphological abnormalities of the sperm flagella (MMAF) phenotype in affected individuals. **A** Hematoxylin and Eosin (H&E) staining of spermatozoa from affected individuals showing multiple flagella defect, including bent, short, and coiled tails. Scale bars, 5 μm. **B** Quantitative assessment of sperm morphology. At least 200 spermatozoa per patient were evaluated and the data are presented as the mean ± SEM. ***, *P* < 0.001 and **, *P* < 0.01; Student’s t-test. **C** Scanning electron microscopy (SEM) of spermatozoa from patient P2 (II:5) revealed typical MMAF defects, including absent, short, bent and coiled sperm flagella. Scale bars, 5 μm

**Fig. 3:**
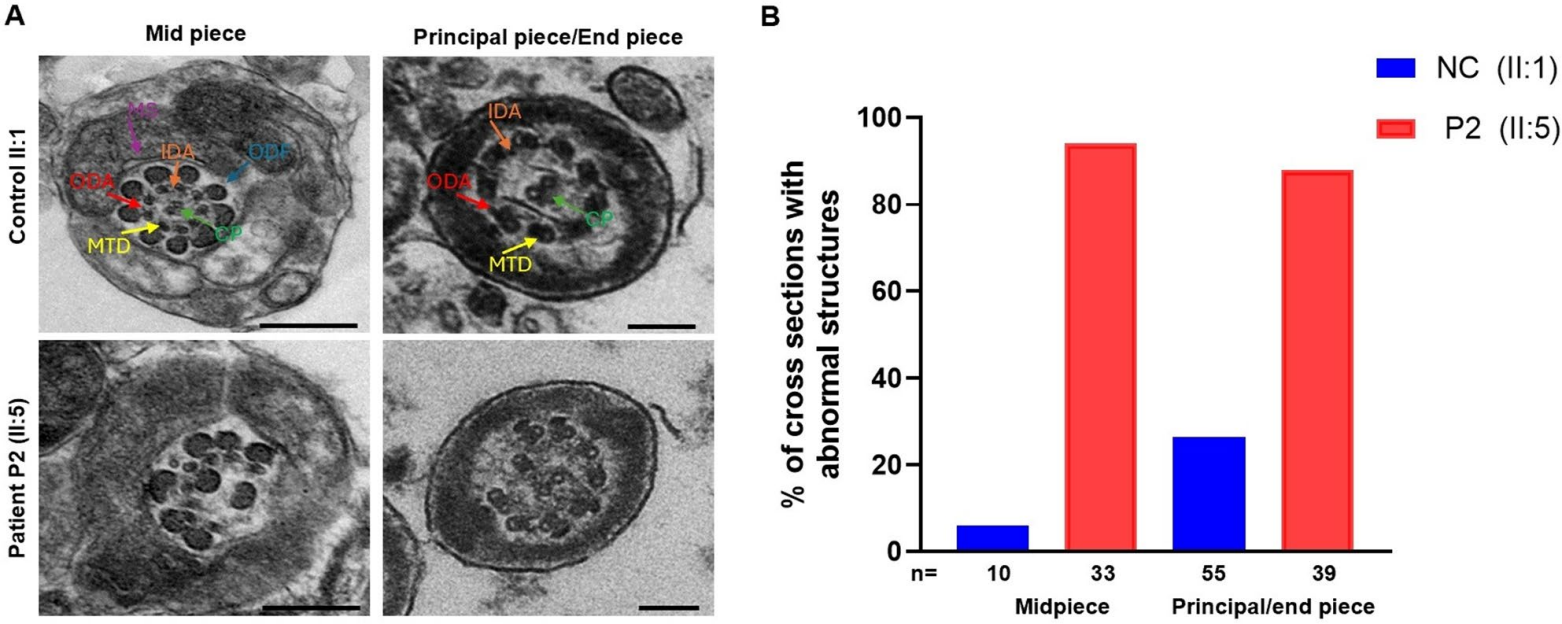
Ultrastructural abnormalities in sperm flagella revealed by transmission electron microscopy. **A** TEM analysis of sperm flagella from patient P2 (II:5) showing severely disorganized or absent axonemal microtubule doublets (DMTs). The axoneme structure in control presents a ‘9 + 2’ microtubules arrangement, including mitochondrial sheath (MS, indicated with plum arrow), central pair of microtubules (CP, indicated with green arrow), outer dense fibers (ODFs, indicated with turquoise arrow), peripheral doublet microtubules (DMTs, indicated with yellow arrow), inner dynein arm (IDA, indicated with orange arrow), and outer dynein arm (ODA, indicated with red arrow) scale bars, 500 nm. **B** Quantification of abnormal axonemal cross-sections in patient P2 (II:5) compared to an unaffected control brother (II:1)

To determine the phenotypic consequences of the *CFAP65* mutation, we assessed clinical and semen parameters of the affected individuals (P1 and P2). Although sperm concentration was within the normal range per WHO guidelines, both individuals exhibited severe asthenozoospermia, with markedly reduced sperm motility. Hematoxylin-eosin (H&E) staining of sperm smears revealed a dramatically decreased proportion of morphologically normal spermatozoa in both patients (3.07 ± 0.07% in P1and 2.67 ± 0.33% in P2), along with a significantly increased frequency of abnormal flagellar phenotypes, including coiled, short, bent, irregular, and absent flagella (Fig. 2A and B). To further investigate ultrastructural defects, we performed scanning and transmission electron microscopy (SEM and TEM) on spermatozoa from the affected patient P2 (II:5) and an unaffected fertile sibling (II:1). SEM analysis showed that normal sperm had a regular oval head and a continuous flagellum with an intact midpiece and tail (Fig. 2C). Spermatozoa from the affected patient exhibited multiple morphological abnormalities, including shortened, coiled, bent, or absent flagella. TEM analysis of longitudinal sections in control sperm revealed a normal “9 + 2” axonemal arrangement-nine peripheral doublet microtubules (DMTs), a central pair complex [34], outer dense fibers (ODFs), and fibrous sheath [35]. In contrast, spermatozoa from P2 showed severe axonemal disorganization, characterized by disrupted or missing DMTs, confirming structural defects consistent with the MMAF phenotype (Fig. 3A and B). To date, several biallelic loss-of-function mutations in *CFAP65* have been reported in patients with MMAF, with variable expression of acrosomal and respiratory phenotypes (Table 2). A summary of all previously reported *CFAP65* mutations and their associated phenotypes is presented in Table 2, highlighting this variability. Importantly, none of the affected individuals in the current study reported chronic respiratory symptoms and had no situs inversus, and chest radiographs showed no abnormalities suggestive of PCD, indicating an isolated sperm-specific phenotype (Supplementary Figure S2).

**Table 2:** Genotype-phenotype correlation of reported *CFAP65* mutations

Variant (DNA & protein level)	Sperm Flagellar Phenotype	Sperm Acrosomal Phenotype	PCD / Respiratory Symptoms	References
c.5341G> T p.E1781X;	Severe MMAF with CPC missing and disorganized FS	Head morphology defects ranging from mild to severe, such as an amorphous, tapered or small head, indicating a defect in sperm head shaping or acrosome formation	Chronic cough, sinusitis, pneumonia, nasal obstruction, pneumonia, recurrent airway infections, bronchitis, rhinosinusitis reported	ADDIN EN.CITE [13]
c.C2284T p.R762X; c.1751delC p. P584fs	Severe MMAF with CPC missing and disorganized FS	Head morphology defects ranging from mild to severe, such as an amorphous, tapered or small head, indicating a defect in sperm head shaping or acrosome formation	Chronic wet cough, nasal obstruction and pneumonia. However, paranasal sinus and chest CT scans were normal without any abnormalities	ADDIN EN.CITE [13]
c. C3021A p. N1007K; c. 5714_5721del P. L1905fs	Not reported	Not reported	No respiratory symptoms	ADDIN EN.CITE [13]
c.5341G> T p.E1781X; c.1775_1776del p.P527Rfs*74 (Compound heterozygous mutation in 1 patient)	MMAF with axonemal and mitochondrial sheath malformations	Yes (acrosome hypoplasia)	(PCD)-like clinical features, with nasosinusitis and tympanitis (Exact test not reported)	ADDIN EN.CITE [36]
c.2675G> A p.W892X	Isolated MMAF (asthenozoospermia), CPs or DMTs missing, disorganized ODFs	No	Assessed by MRI/CT and patient exhibited no PCD symptoms	ADDIN EN.CITE [37]
*Cfap65*^-/-^ mouse	loss of the C2a projection in ciliary/flagellar axonemes, severe MMAF	hydrocephalus	No PCD symptoms	ADDIN EN.CITE [38]
c.582_587delinsCG; p.Q194Hfs*4	MMAF (asthenozoospermia), axonemal disorganization with MTDs absence/disrupted	No	Assessed by chest X-ray, no chronic cough or pneumonia-like symptoms reported in patients	Current study

*Abbreviations*: *MMAF* Multiple morphological abnormalities of the sperm flagella, *CP* Central pair, *CPC* Central pair complex, *FS* Fibrous sheath, *DMTs* Doublet microtubules, *ODFs* Outer dense fibers, *PCD* Primary ciliary dyskinesia, *MRI* Magnetic resonance imaging, *CT* Computed tomography

## Discussion

Effective sperm motility is crucial for natural fertilization, and structural abnormalities of the sperm flagellum are a well-established cause of male infertility, particularly in individuals with multiple morphological abnormalities of the sperm flagella (MMAF). Although pathogenic variants in several genes-including *DNAH1*, *CFAP43*, *CFAP44*, and *ARMC2*-have been implicated in MMAF [9, 39, 40], the genetic etiology remains unknown in approximately 40–50% of cases. This highlights the need for continued research to identify additional causative genes and further elucidate the molecular basis of MMAF.

In this study, we identified a novel homozygous frameshift mutation in *CFAP65* (c.582_587delinsCG) in two infertile male brothers from a consanguineous Pakistani family. This mutation, located in exon 4, was confirmed by Sanger sequencing and shown to follow an autosomal recessive inheritance pattern. Transmission electron microscopy of spermatozoa from affected individuals revealed isolated axonemal disorganization-specifically, loss of microtubule doublets (DMTs), without acrosomal defects. This contrasts with previously described *CFAP65* variants that disrupt acrosome-manchette interactions during spermiogenesis [23, 41], suggesting a domain-specific functions organization: the transmembrane helix domain appears essential for axonemal integrity, whereas other regions of the protein may regulate acrosomal development. Further functional studies are needed to elucidate the precise molecular mechanisms underlying these domain-specific roles.

*CFAP65*, located on 2q35, was previously identified in human spermatozoa through mass spectrometry-based proteomic analysis [42]. It encodes a putative transmembrane protein with two conserved domains: the ASH (ASPM, SPD-2, Hydin) domain and the MSP (major sperm protein) domain. The ASH domain is associated with proteins involved in cilia, flagella, centrosome and the Golgi apparatus, while the MSP domain mediates protein-protein interaction and contributes to cytoskeletal organization and intracellular signaling [43]. In the present study, the frameshift mutation likely disrupts the transmembrane region, impairing the structural assembly of the axoneme, particularly DMT organization, thereby leading to the severe MMAF phenotype observed in the affected individuals.

Prior studies show *CFAP65* serves as a scaffold protein at the nuclear surface, facilitating acrosome anchoring and manchette complex transport during spermatid elongation [44, 45]. Notably, *CFAP65*’s transmembrane domain is critical for basal body docking and F-actin apical enrichment in multiciliogenesis [46], but its specific function in sperm flagellar axoneme organization remains poorly understood. Most research on *CFAP65* has primarily examined its involvement in male infertility. Studies have shown that biallelic mutations in this gene cause both acrosome malformation and multiple sperm flagellar abnormalities, ultimately resulting in severe asthenoteratozoospermia [13, 23, 47]. Our findings indicate that disruption of the transmembrane region selectively compromises axonemal structure without overt acrosomal defects, further supporting domain-specific functionality.

Although *CFAP65* is expressed in several ciliated tissues, our patients exhibited no clinical symptoms. Notably, previous research has reported broader phenotypes: one study identified biallelic *CFAP65* mutations associated with severe asthenoteratozoospermia due to acrosome hypoplasia and flagellar malformations [13], while *Cfap65* knockout mice displayed profound sperm flagellar defects (MMAF) and a high incidence of hydrocephalus, yet no significant impairment of respiratory cilia [47]. The absence of extra-gonadal symptoms in our patients suggests that the identified mutation primarily affects sperm flagella, although the possibility of subclinical ciliary dysfunction cannot be entirely excluded. Further studies involving functional assays and multi-organ phenotyping will be necessary to elucidate the full spectrum of *CFAP65*-related ciliopathies.

This study expands the phenotypic and mutational landscape of *CFAP65*-associated male infertility by identifying a novel domain-specific frameshift mutation that selectively disrupts axonemal integrity. Our work highlights the importance of mutation localization in predicting phenotypic outcomes and provides a framework for precision diagnostics in consanguineous populations. Future research should aim to elucidate the precise molecular mechanisms by which *CFAP65* regulates flagellar assembly and investigate its potential involvement in ciliary function beyond the male reproductive system.

## Conclusion

This study identifies a novel homozygous frameshift mutation in the *CFAP65* gene, which causes MMAF in two infertile brothers. The mutation disrupts the protein’s transmembrane domain, leading to isolated axonemal disorganization without acrosomal defects. This highlights a domain-specific function for *CFAP65*, wherein this region is critical for flagellar integrity. The findings expand the genetic and phenotypic spectrum of MMAF and emphasize the importance of mutation location in predicting clinical outcomes, providing a basis for improved diagnostics and future research into *CFAP65*’s role in ciliary function.

## Supplementary Information

Supplementary Material 1.

## Data Availability

All the data presented in the article and its supplementary materials support the conclusions drawn in this study. Additional data can be provided by the corresponding author upon request.
