# Supplementary material for: A novel homozygous frameshift mutation in CFAP65 is associated with multiple morphological abnormalities of sperm flagella in a consanguineous Pakistani family
Source: Basic Clin Androl. 2026 Jul 20;36:22. doi: 10.1186/s12610-026-00319-z (PMC13383139; doi:10.1186/s12610-026-00319-z)

**Supplementary Figure S1: Genome-wide linkage and whole-exome sequencing (WES) analysis.**

(A) Genome-wide linkage analysis identified a candidate region with a LOD score > 0.5 under a recessive model.

(B) Homozygosity mapping of affected individuals P1 (II:3) and P2 (II:5) revealed shared homozygous regions, with the most significant regions highlighted in red. An asterisk (*) indicates the location of *CFAP65*.

(C) Schematic of the WES data filtration pipeline used to prioritize candidate variant.


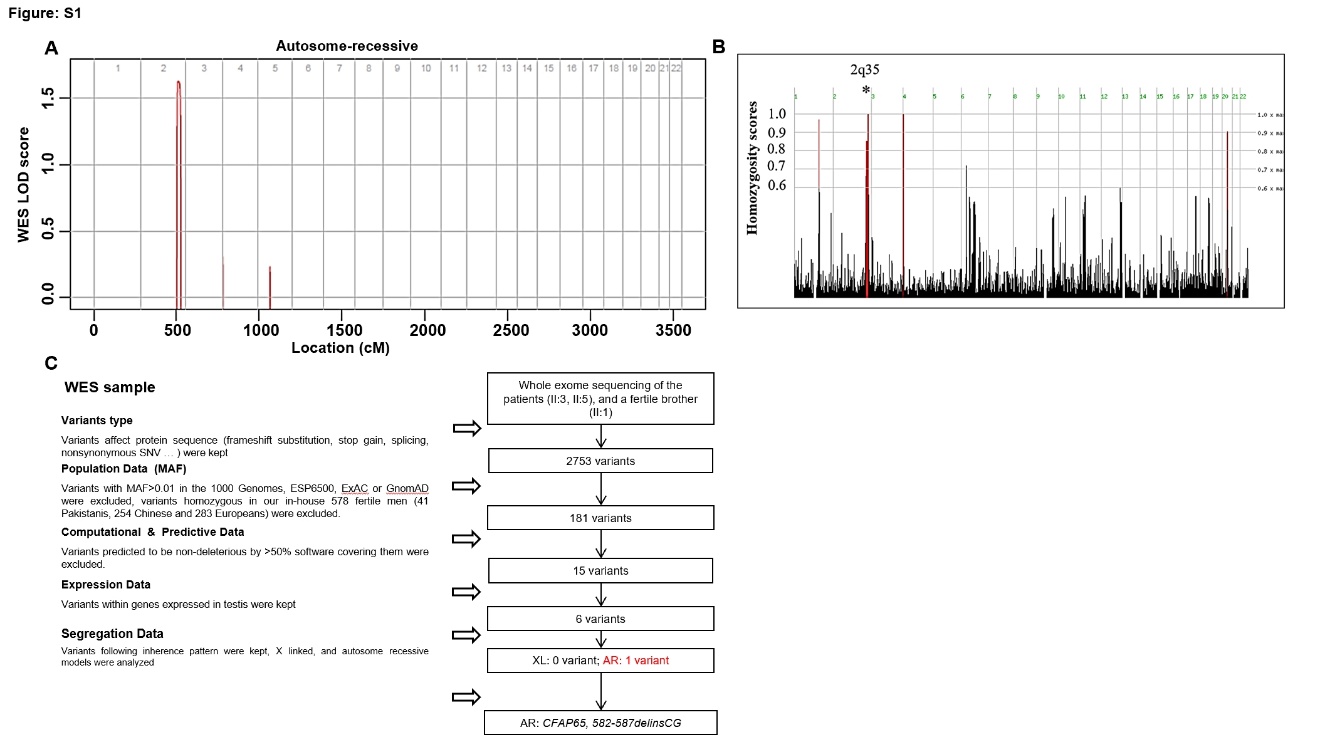


**Supplementary Figure S2: Chest radiography showing no evidence of primary ciliary dyskinesia (PCD)-related lung pathology in affected individuals.**

**C**hest X-ray images of patients P1 (II:3) and P2 (II:5) reveal clear lung fields with no signs of bronchiectasis, atelectasis, chronic infiltrates, or other abnormalities that would indicate PCD or chronic respiratory disease.


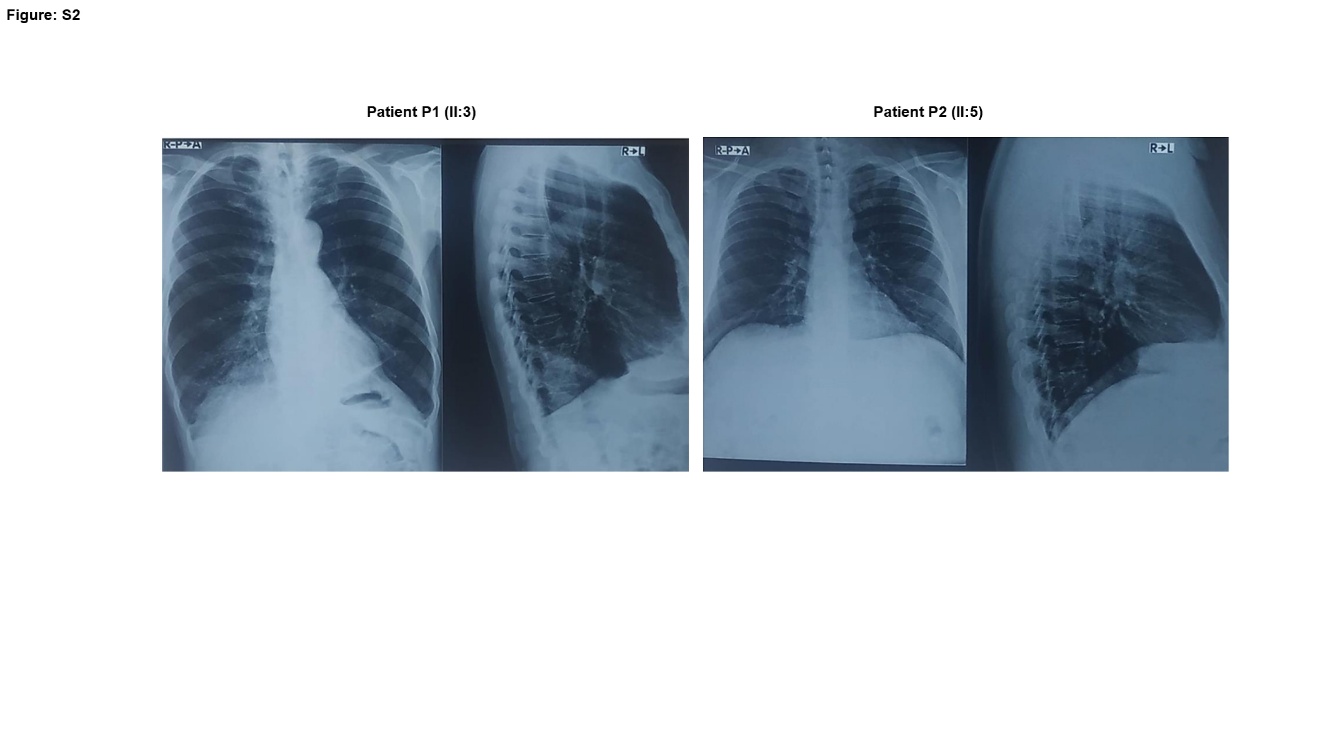


**Supplementary Figure S3: Expression of *CFAP65* in human testicular tissue.**

Gene expression overview from the FANTOM5 dataset (Human Protein Atlas) showing *CFAP65* is highly expressed in the human testicular tissue, with particularly strong expression observed in both early and late spermatid.


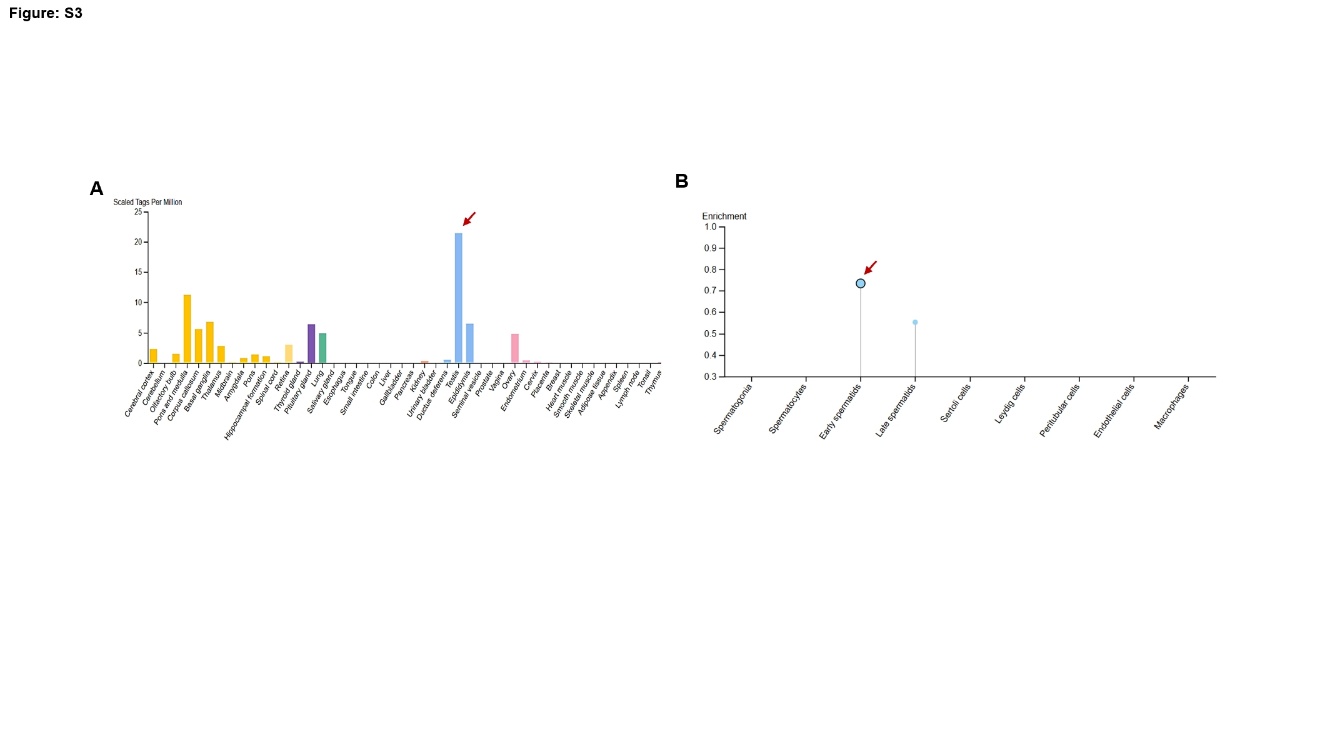

Supplement: Supplementary file 1 — Supplementary Material 1. [file 12610_2026_319_MOESM1_ESM.docx]
